# Theoretical Study on Metasurfaces for Transverse Magneto-Optical Kerr Effect Enhancement of Ultra-Thin Magnetic Dielectric Films

**DOI:** 10.3390/nano11112825

**Published:** 2021-10-25

**Authors:** Jing Chen, Guohua Wu, Ping Gu, Yumei Tang, Chun Yang, Zhendong Yan, Chaojun Tang, Zhengqi Liu, Fan Gao, Pinggen Cai

**Affiliations:** 1 College of Electronic and Optical Engineering, Nanjing University of Posts and Telecommunications, Nanjing 210023, China; jchen@njupt.edu.cn (J.C.); 1219023306@njupt.edu.cn (G.W.); guping@njupt.edu.cn (P.G.); 1219023305@njupt.edu.cn (Y.T.); 1018020724@njupt.edu.cn (C.Y.); 2 College of Science, Nanjing Forestry University, Nanjing 210037, China; zdyan@njfu.edu.cn; 3 College of Science, Zhejiang University of Technology, Hangzhou 310023, China; fangao@zjut.edu.cn (F.G.); caippgg@zjut.edu.cn (P.C.); 4 Jiangxi Key Laboratory of Nanomaterials and Sensors, Provincial Key Laboratory of Optoelectronic and Telecommunication, College of Physics and Communication Electronics, Jiangxi Normal University, Nanchang 330022, China

**Keywords:** metasurface, magnetic resonance, strong coupling, transverse magneto-optical Kerr effect

## Abstract

We study how to enhance the transverse magneto-optical Kerr effect (TMOKE) of ultra-thin magnetic dielectric films through the excitation of strong magnetic resonances on metasurface with a metal nanowire array stacked above a metal substrate with an ultra-thin magnetic dielectric film spacer. The plasmonic hybridizations between the Au nanowires and substrate result in magnetic resonances. The periodic arrangement of the Au nanowires can excite propagating surface plasmon polaritons (SPPs) on the metal surface. When the SPPs and the magnetic resonances hybridize, they can strongly couple to form two strong magnetic resonances, which are explained by a coupled oscillator model. Importantly, benefitting from the strong magnetic resonances, we can achieve a large TMOKE signal up to 26% in the ultra-thin magnetic dielectric film with a thickness of only 30 nm, which may find potential applications in nanophotonics, magnonics, and spintronics.

## 1. Introduction

Recently, obtaining the enhancement of magnetic fields in the visible frequency has become as important in nanophotonics as obtaining the enhancement of electric fields, stemming from many applications, for example, magnetic sensors or magnetic nonlinearity [1,2,3,4,5,6,7,8,9]. In the interactions between light and matter, the magnetic contribution of light can be neglected generally because it is too weak [10]. Thus, it is very important to seek new mechanisms to enhance the magnetic field component of light. Like the electric plasmonic resonances, the interactions between the magnetic resonances and other optical resonances in metasurfaces have been proposed to enhance the magnetic fields, which are however still studied rarely. For instance, by coupling with some narrow-band optical resonances such as the lattice surface modes [11,12], waveguide modes [13,14], or Bloch surface waves [15], the strong magnetic resonances have been obtained on metasurfaces, which exhibit a huge enhancement of magnetic fields. In recent years, we have theoretically shown that if such a coupling appears between the SPPs and the magnetic resonances in periodic arrays of metallic nanowires on an ultra-thin dielectric film spacer deposited on a very thick metal film, a 2143-fold magnetic field enhancement can be achieved in the ultra-thin dielectric film [16].

Usually, the transverse magneto-optical Kerr effect (TMOKE) can be calculated by the variation in the reflected intensity for p-polarized light, under the external static magnetic field with a direction changed from the saturated state +M_s_ to −M_s_, where M_s_ is the saturation magnetization [17]. The realization of a large TMOKE response is now drawing increasing attention, owing to some practical applications such as 3D imaging [18], magnonics [19,20], and magneto-optical data storage [17]. In particular, achieving a large TMOKE response in ultra-thin magnetic dielectric films is important for many potential applications in nanophotonics and spintronics [21,22]. However, the natural TMOKE signal of the ultra-thin magnetic films is quite weak, and its detection is very difficult. Thus, seeking new methods to enhance the TMOKE signal of ultra-thin magnetic films is very important. It is well known that plasmonic resonances are able to enhance TMOKE response in magneto-plasmonic crystals [23,24,25]. Very recently, the enhanced TMOKE signal was also observed for plasmonic metal covered thick magnetic films with a thickness larger than 100 nm [26,27]. However, it is still challenging to obtain giant TMOKE enhancement in magnetic dielectric films with a thickness smaller than 50 nm.

In this work, we will study theoretically the TMOKE enhancement in magnetic dielectric films with a thickness smaller than 50 nm by strong magnetic resonances on simple metasurfaces. The metasurfaces are composed of a metal nanowire array, a metal substrate, and an ultra-thin magnetic dielectric film spacer between them. The plasmonic hybridizations between the metallic nanowires and substrate result in magnetic resonances. The metal nanowire array is able to excite SPPs propagating on the surface of the metal substrate. When the positions of the SPPs and the magnetic resonances are close by tuning the array period, they strongly couple to form two strong magnetic resonances. Furthermore, thanks to the huge magnetic field enhancement at the strong magnetic resonances, a large TMOKE signal that exceeds 26% can be achieved in the ultra-thin magnetic film, which has a thickness of only 30 nm. Our work may have potential applications to designing the high-efficiency magneto-optical devices.

## 2. Methods

**Figure 1:**
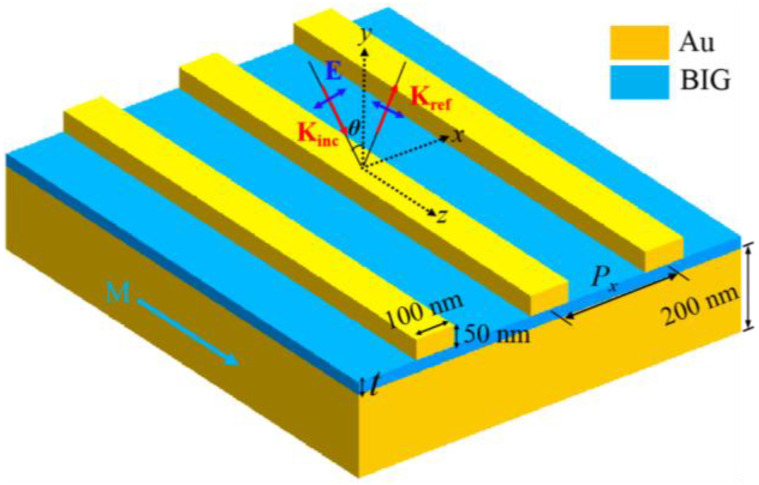
Schematic of metasurfaces for the TMOKE enhancement of ultra-thin BIG films. Au nanowires are infinite along the *z* direction and periodically arranged along *x* direction, which have a cross section of 100 × 50 nm. The ultra-thin BIG magnetic film has a thickness of *t*. The illumination source is p-polarized and propagates in the *x*-*y* plane. The external magnetization vector, M, is parallel to the *z* axis.

Figure 1 shows schematically the designed metasurface for the TMOKE enhancement of an ultra-thin magnetic dielectric film. It consists of an Au nanowire array, an Au film of 200 nm thickness, and a magnetic dielectric film of bismuth iron garnet (BIG). The Au nanowires have the same size of 100 × 50 nm, which are infinite along the *z* direction and periodically arranged along the *x* direction. The ultra-thin BIG magnetic film has a thickness of *t*. For producing a transverse magneto-optical Kerr effect, the p-polarized light is obliquely incident on the surface of the metasurfaces, and the incident angle is *θ* = 8^0^. The additional magnetization vector M is perpendicular to the incidence plane, i.e., the *x-y* plane. The well-known software package (Comsol Multiphysics) is used to calculate the reflection spectra, the magnetic fields, and the TMOKE responses. In our numerical calculations, the frequency-dependent relative permittivity of Au has a Drude model *ε_gold_* = 1 − *ω_p_^2^*/[*ω*(*ω* + *iω_c_*)]. The plasma frequency *ω_p_* is 1.367 × 10^16^ rad/s and the collision frequency *ω_c_* is 4.084 × 10^13^ rad/s [28]. Under the external applied magnetic field with the magnetization (M) along the *z* axis, the dielectric permittivity tensor of the ultra-thin BIG magnetic film can be written as:

$$\varepsilon =\left(\begin{array}{ccc} \varepsilon_{0} & ig & 0\\ -ig & \varepsilon_{0} & 0\\ 0 & 0 & \varepsilon_{0} \end{array}\right)$$

where *ε*_0_ = 6.76 + 0.3i is the dielectric function of the BIG film in the case of no magnetization, and *g* = 0.016 − 0.008i when taking into account the magneto-optical activity [27]. The BIG is a magnetic medium with excellent magneto-optical properties and low loss, and recently the BIG has been widely applied by researchers to study magneto-optical effects [27].

The TMOKE signal is quantified as the relative intensity change of the reflected light for the magnetization M to be reversed:

(1) $$\mathrm{TMOKE}=\frac{R(+M)-R(-M)}{R(0)}$$

where *R*(+M) and *R*(−M) are the reflectance of the p-polarized incident light for the positive and negative external applied magnetic fields, respectively, and *R*(0) denotes the reflectivity when the metasurfaces are demagnetized. The designed metasurfaces can be experimentally realized by advanced nanotechnology: the BIG film is firstly deposited on the surface of the Au substrate by thermal evaporation [29]. Then, by the electron beam lithography the Au nanowire array is fabricated on the BIG film [30,31]. The magneto-optical Kerr effect measurements can be conducted with a Kerr spectrometer consisting of a broadband supercontinuum laser (NKT SuperK EXW-12 with acousto-optical filter) [32].

## 3. Results and Discussion

**Figure 2:**
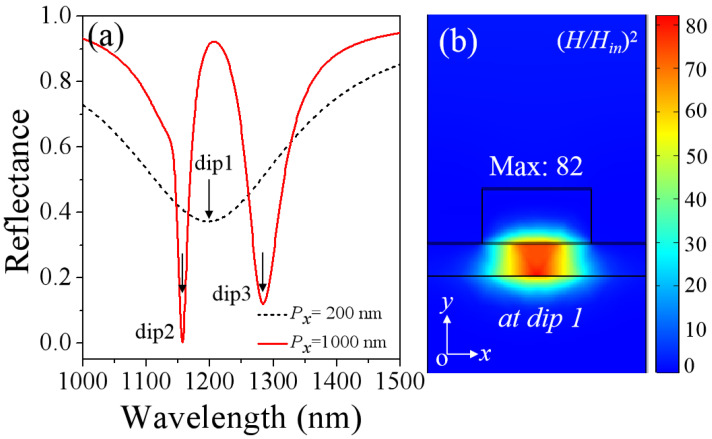
(**a**) The calculated reflection spectra of the designed metasurfaces with *P_x_* = 200 (black dotted line) and 1000 nm (red line), with the thickness of the BIG film *t* = 30 nm and the incident angle of *θ* = 8^0^. (**b**) The intensity distributions of normalized magnetic field (*H*/*H_in_*)^2^ on the *xoy* plane for the dip 1 resonance. The boundaries of different materials are shown by the black solid lines.

To understand the nature of the designed metasurfaces, we first study the optical properties of the metasurfaces when the alternate magnetic field is not applied. Figure 2a presents the reflection spectra of the metasurfaces with a period *P_x_* = 200 and 1000 nm. The thickness of the BIG film is *t* = 30 nm and the incident angle is *θ* = 8^0^. A quite broad reflection dip (marked as dip 1) at *λ_1_* = 1198 nm is observed in Figure 2a, when the array period *P_x_* = 200 nm (black dotted line). The broad reflection dip is due to magnetic resonances in individual Au nanowires stacked above the thick Au substrate with the 30 nm BIG film spacer [33,34]. Figure 2b gives the magnetic field intensity distributions at dip 1. Obviously, the magnetic fields are localized highly within the BIG film, which is the field distribution property of a magnetic resonance [33,34]. When the array period *P_x_* = 1000 nm, the broad reflection dip will split into two relatively narrower strong magnetic resonances [marked as dip 2 (1151 nm) and dip 3 (1267 nm)], as exhibited by the red line in Figure 2a.

**Figure 3:**
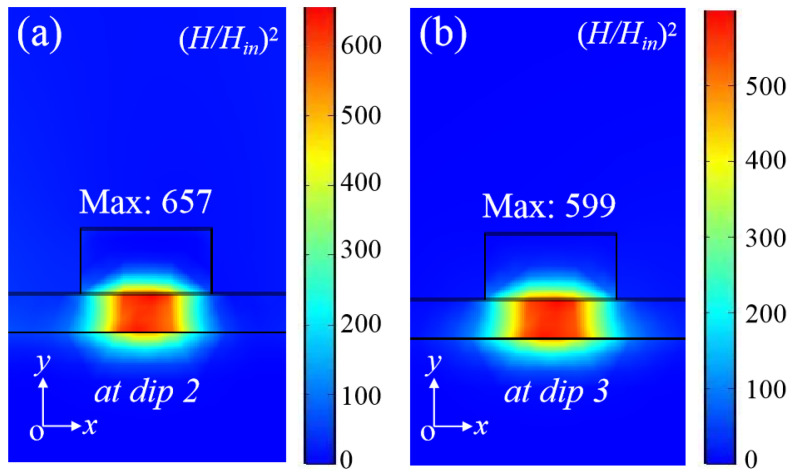
And the intensity distributions of normalized magnetic field (*H*/*H_in_*)^2^ on the *xoy* plane for the dip 2 (**a**) and dip 3 (**b**) resonances. The boundaries of different materials are shown by the black solid lines.

In Figure 3a,b, we present the normalized magnetic field distributions for the resonances at dip 2 and dip 3, respectively. There is the same field distribution property as at dip 1. However, the maximum magnetic fields become much stronger at dip 2 and dip 3, which are 657- and 599-times of the incident magnetic field, and are 8.01- and 7.30-times of the corresponding value at dip 1, respectively.

To reveal the physical origin of the two relatively narrower strong magnetic resonances in Figure 2a, we have employed the coupling model of double oscillators to explain their positions for different period *P_x_* (This strong coupling leads to the formation of two mixed modes, i.e., the highand low-energy states E+,− ) [35]:

(2) $$E_{+,-}=(E_{\mathrm{MR}}+E_{\mathrm{SPPs}})/2\pm \sqrt{\Delta /2+{\left(E_{\mathrm{MR}}-E_{\mathrm{SPPs}}\right)}^{2}/4}$$

**Figure 4:**
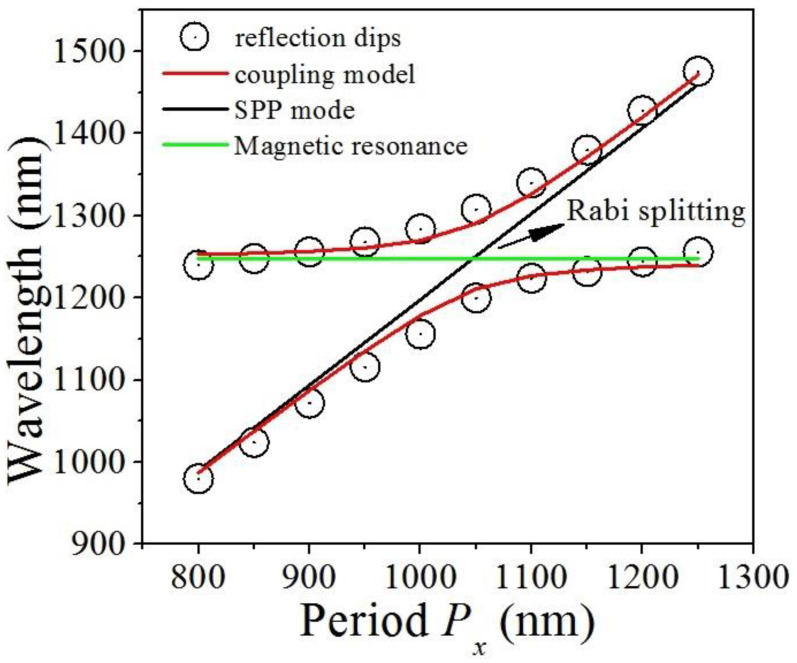
The positions of reflection dips (dip 2 and dip 3 in Figure 2) for different periods. The position (horizontal green line) of the magnetic resonance and the position (black diagonal line) of the SPPs are also shown.

Here, *E*_MR_ and *E*_SPPs_ are the energies of magnetic resonances and SPPs, respectively. The physical quantity of Δ is the coupling strength between magnetic resonances and SPPs. In Figure 4, the open black circles are the practical positions of the two relatively narrower strong magnetic resonances, which are taken from the calculated reflection spectra for different period *P_x_*. The two branches of red lines are the corresponding results predicted by the coupling model with a coupling strength of Δ = 200 meV. Evidently, this coupling model is able to reproduce excellently the practical positions. This suggests that the two relatively narrower strong magnetic resonances in Figure 2a arise from the coupling of the SPPs and the magnetic resonances in metasurfaces. Besides, in Figure 4 at the cross between the SPPs and the magnetic resonance, the two relatively narrower strong magnetic resonances present an obvious anticrossing. Therefore, the interesting physical phenomenon similar to Rabi splitting appears, which is a key characteristic of the strong coupling between the SPPs and the magnetic resonance [13,15,35,36].

**Figure 5:**
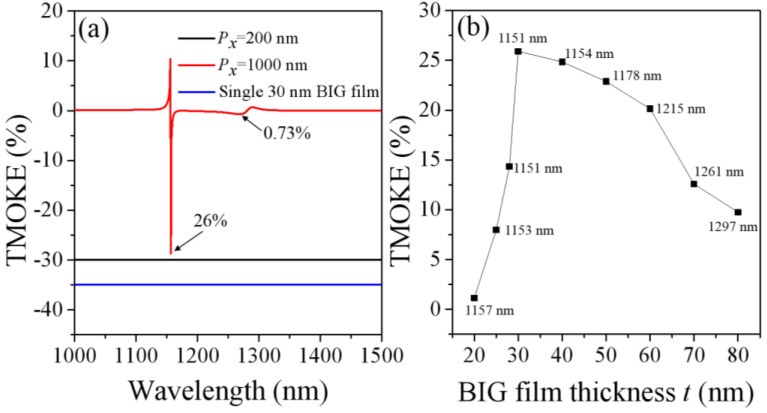
(**a**) The TMOKE spectra for the designed metasurface with the period *P_x_* = 1000 nm (red line). For the comparison, the TMOKE spectrum for the designed metasurface with the period *P_x_* = 200 nm and the single 30 nm BIG film are also given by the black and blue line, which are shifted vertically for better visibility. (**b**) The maximum intensity of the TMOKE signal for metasurface with the different BIG film thickness (*t*), with the period *P_x_* = 1000 nm.

Under the strong magnetic resonances, the magnetic fields is significantly enhanced and highly confined in the ultra-thin BIG film, which is favorable for TMOKE enhancement. Next, we investigated in detail the TMOKE response with the designed metasurfaces. Figure 5a presents the TMOKE spectra for the metasurfaces with the period *P_x_* = 200 and 1000 nm, in which the TMOKE spectrum of the single 30 nm BIG film is also shown for the comparison. As can be seen, because of the strong magnetic resonances, the metasurface with the period *P_x_* = 1000 nm has a large TMOKE enhancement with amplitude of 26%, which is much higher than that of the prism-based sandwich architectures [23,27]. For sandwich configurations, the TMOKE signal usually has an order of magnitude of 0.1–10% [37]. After this optimization of the coupling of the SPPs and the magnetic resonances, we find the optimal BIG film thickness for the highest TMOKE values. Figure 5b shows the maximum intensity of the TMOKE signal of the metasurfaces with the different BIG film thickness (*t*), where the period of the nanowire array *P_x_* is 1000 nm. When the BIG thickness is increased from 20 to 30 nm, the TMOKE intensity initially becomes larger, but it will be deteriorated with the BIG thickness further increased from 30 nm to 80 nm. When the BIG thickness *t* is only 30 nm, the TMOKE signal reaches to a maximum of 26%, which arises from strong magnetic resonances in the simple metasurface. The TMOKE signal achieved now is among the highest level of the recently reported magneto-plasmonic crystals [38]. It can be seen that when the thickness of the BIG is 30 nm, the TMOKE signal is the strongest. Therefore, in the previous part, we studied the case of BIG thickness of 30 nm in detail.

## 4. Conclusions

In summary, we have studied theoretically the TMOKE enhancement of ultra-thin magnetic films, originating from strong magnetic resonances in simple metasurfaces. The ultra-thin magnetic dielectric film is sandwiched between the Au nanowire array and the Au substrate. The plasmonic hybridizations between the individual Au nanowires and the Au substrate lead to magnetic resonances. The periodic arrangement of the Au nanowires can excite SPPs propagation in the Au substrate. When the SPPs and the magnetic resonances couple, they can form two strong magnetic resonances. Moreover, owing to the large enhancement of magnetic fields at the strong magnetic resonances, a large TMOKE signal has been achieved in the ultra-thin magnetic film with a thickness of only 30 nm, which may find potential applications in nanophotonic and spintronic devices.

## Data Availability

The study did not report any data.
